# The complete chloroplast genome of *Butomus umbellatus* L. and its phylogenetic position

**DOI:** 10.1080/23802359.2019.1678435

**Published:** 2019-10-21

**Authors:** Zhaoping Yang, Luxian Liu

**Affiliations:** a College of Life Sciences, Tarim University, Alaer, P. R. China;; b Key laboratory of Plant Stress Biology, School of Life Sciences, Henan University, Kaifeng, P. R. China

**Keywords:** Chloroplast genome, phylogenomics, *Butomus umbellatus*

## Abstract

*Butomus umbellatus*, which belongs to Butomaceae, is a plant species typical of littoral communities of river and stream shores. Here, we reported and characterized the complete chloroplast (cp) genome of *B. umbellatus* and analyzed its phylogenetic position based on the complete cp genome sequences of 10 species within the core Alimatales. The cp genome is 158,107 bp in length, which consists of a large single-copy region (LSC, 88,140 bp； GC content: 34.8%), a small single-copy region (SSC, 19,695 bp; GC content: 30.1%), and a pair of inverted repeat regions (IRs, 25,136 bp; GC content: 42.9%). It harbours 113 unique genes, including 79 protein-coding genes, 30 transfer RNA genes, and 4 ribosomal RNA genes with an overall GC content of 36.8%. The phylogeny inference showed that *Butomaceae and Hydrochariaceae* formed a high supported clade, which was sister to Alismataceae. This result was similar to the floral development from petaloid Alismatales, and also similar to the phylogenetic studies of Alismales based on 83 plastid genes, but with much higher bootstrap support.

*Butomus umbellatus* is one species of Butomaceae, which occurs in shallow standing waters (fishponds or other reservoirs, temporarily flooded field depressions), in small streams or on river banks, mostly on sites with fluctuating water level (Iwamoto et al. 2018). This species usually distributes in North Europe, Asia, and North America. However, in North America, *B. umbellatus* gradually became an aggressive, invasive, aquatic plant, and it is displacing many native aquatic/wetland plants (Turnage et al. 2019). In recent years, studies on *B. umbellatus* focus on reproduction biology. Within three ‘petaloid’ families of Alimatales, the inner petals of Butomaceae and Hydrocharitaceae are not delayed, but Alismataceae is delayed (Iwamoto et al. 2018). The question is whether Butomaceae is more closely related to Hydrocharitaceae than to Alismataceae? Here, we firstly reported the complete chloroplast genome characteristics of *B. umbellatus* and reconstructed its phylogenetic relationship.

Leaf samples of *B. umbellatus* were collected from the Irtysh riverside, Beitun city, Xinjiang, China. The corresponding voucher herbarium specimens (Yang2017005) were deposited at the Herbarium of Tarim University (TAU). Total DNA was extracted from the silica-gel dried leaf tissue using DNA Plantzol Reagent (Invitrogen, Carlsbad, CA, USA), following the manufacturer’s protocol. Then, raw reads were obtained by next-generation sequencing, conducting on the Illumina Hiseq Platform (Illumina, San Diego, CA, USA). The plastome was assembled via NOVOPlasty (Dierckxsens et al. 2017) and deposited into GenBank (accession number: MN484598), with cp genome of *Elodea canadensis* (GenBank accession number: NC_018541) as a reference. The annotation was performed using Geneious 11.0.5 (Biomatters Ltd., Auckland, New Zealand) following description in Liu et al. (2017). The cp genome of *B. umbellatus* is 158,107 bp in length, which consists of a large single-copy region (LSC, 88,140 bp；GC content: 34.8%), a small single-copy region (SSC, 19,695 bp; GC content: 30.1%), and a pair of inverted repeat regions (IRs, 25,136 bp; GC content: 42.9%), similar structure with most of angiosperm species. It harbours 113 unique genes of *B. umbellatus* plastome, including 79 protein-coding genes, 30 transfer RNA genes, and 4 ribosomal RNA genes with an overall GC content of 36.8%.

The phylogeny of 10 Alimatales species was reconstructed based on the complete cp genome sequences, using both maximum likelihood (ML) and Bayesian inference (BI) methods, with one outgroup taxa (*Symplocarpus renifolius*, Caryophyllaceae). We implemented these methods on CIPRES Science Gateway version 3.3 (Miller et al. 2010). RAxML-HPC version 8.2.12 (Stamatakis 2014) and XSEDE version 3.2.6 (Ronquist and Huelsenbeck 2003) were used for building ML and Mrbayes trees, respectively. Consequently, ML and BI analyses generated the same tree topology (Figure 1). *Butomaceae* and *Hydrochariaceae* formed a high-supported evolutionary clade, and this clade was sister to Alismataceae. This result was similar to the floral development from petaloid Alismatales and also similar to the phylogenetic studies of Alismales based on 83 plastid genes (Iwamoto et al. 2018), but with much higher bootstrap support (Ross et al. 2016). Both floral development and phylogenetic studies support that Butomaceae is more closely related to Hydrocharitaceae.

**Figure 1.**
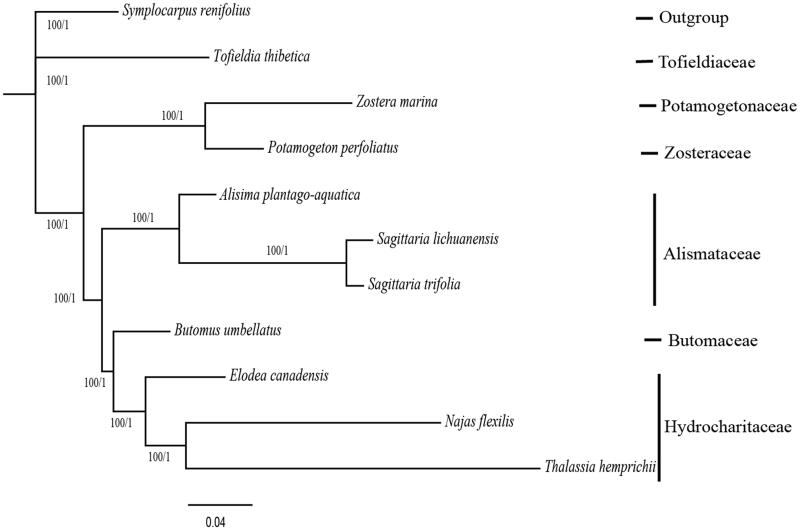
Molecular phylogeny of Core Alismatales based on the complete chloroplast genomes of 10 taxa, with *Symplocarpus renifolius* (Acoraceae) as the outgroup. The accession numbers are listed as below: *Alisma plantago-aquatica* (MK090659), *Elodea canadensis* (NC_018541), *Najas flexilis* (NC_021936), *Potamogeton perfoliatus* (NC_029814), *Sagittaria lichuanensis* (NC_029815), *Sagittaria trifolia* (MK090658), *Symplocarpus renifolius* (NC_033970), *Thalassia hemprichii* (KT899950), *Tofieldia thibetica* (KT899950), and *Zostera marina* (NC_036014). Relative branch lengths are indicated. Numbers above the lines represent ML bootstrap values/BI posterior probability.
